# Examining the Relationship Between Social Vulnerability and Animal Shelter Intakes and Outcomes: Patterns and Implications

**DOI:** 10.3390/ani14223166

**Published:** 2024-11-05

**Authors:** Sue M. Neal, Tom Kremer

**Affiliations:** 1 Department of Political Science, Arkansas State University, Jonesboro, AR 72401, USA; 2 Human Animal Support Services, Austin, TX 78703, USA; tom.kremer@americanpetsalive.org

**Keywords:** animal sheltering, animal surrender, equity, animal adoptions, euthanasia

## Abstract

(1) Background: This study investigates the impact of social vulnerability, as quantified by the Social Vulnerability Index (SVI), on animal shelter intakes and adoptions, and the flow of animals between SVI groups at seven shelters within the United States. Understanding how social vulnerability influences these factors is crucial for developing targeted interventions to improve shelter outcomes and community support programs. (2) Methods: The research analyzed intake and adoption data from multiple animal shelters to assess the relationship between the social vulnerability at intake and adoption locations based on Census Tract obtained from addresses, focusing on comparison across strays, seized animals, and owner surrenders. Effect sizes were calculated to determine the magnitude and practical implications of these relationships. The study also examined the distribution of outcomes and reasons for animal surrender in relation to SVI levels. (3) Results: The analysis found that animals tended to enter shelters from areas of higher vulnerability while being adopted out to homes from all levels of vulnerability. Stray animals and those seized by authorities were more likely to come from high-SVI areas and showed a stronger disparity between intake and adoption SVI levels than owner surrenders. Racial/ethnic minority status and socioeconomic status were the components of the SVI that produced the strongest differences between intake and adoption vulnerability levels. Adoptions, however, were evenly distributed across different SVI levels, suggesting equitable access to adoption services. The study also identified that animals from high-SVI areas were not euthanized at higher rates compared to those from lower-SVI areas. (4) Conclusions: The findings highlight the need for further investigation into the high numbers of stray adult dogs in areas of communities that are most socially vulnerable as well as programs to address the volume of young animals originating from these areas. The equitable distribution of adoptions across SVI levels suggests that shelters are effectively engaging with socioeconomically and racially/ethnically diverse communities. Future research should focus on exploring the dynamics of stray animal intakes and enforcement patterns to enhance community and animal welfare initiatives.

## Simple Summary

This study looked at how social and economic challenges, measured by the Social Vulnerability Index (SVI), affect animals entering and being adopted from seven shelters in the United States. We found that areas with higher social vulnerability, often characterized by more racial and ethnic minorities and lower income, had more animals coming in as strays or being seized by authorities, while adoptions were fairly evenly spread across vulnerability groups. Stray animals, in particular, were more common from high-vulnerability areas compared to owner surrenders, which were less influenced by social vulnerability. Interestingly, animals from these high-vulnerability areas were not more likely to be euthanized than those from other areas. This suggests that shelters in this study are engaging with adopters across diverse community members. Recommendations include further research into the high volume of stray adult dogs as well as spay/neuter and Trap-Neuter-Vaccinate-Return programming targeted in areas of the community that are the most socially vulnerable to address the volume of young animals.

## 1. Introduction

Animal shelters in the United States continue to focus on ways to increase the proportion of live outcomes for animals in their care. Researchers have primarily explored questions of “who and why” of animal surrender and animal adoption in an attempt to better balance these two sides of the flow of animals through the shelter system. A significant portion of animal relinquishment is attributed to the personal circumstances of the owners. Studies indicate that owner-related factors account for the majority of reasons for surrendering pets, with issues such as poor health, family changes, and lifestyle adjustments being prominent [1,2,3]. For instance, a study found that 75% of relinquishments for cats and 74% for dogs were due to owner-related reasons, with poor owner health being the most common [1]. Additionally, changes in family structure, such as the arrival of children or new partners, often lead to the decision to surrender pets [4].

Individuals from lower socioeconomic backgrounds may be more prone to needing to rehome their pets, particularly cats and dogs, due to a variety of factors including financial constraints, housing instability, and lack of access to veterinary care. Socioeconomic status plays a significant role in pet relinquishment. Lower-income households may struggle to afford veterinary care, training, and other resources necessary for pet guardianship [5,6]. A lack of awareness about available support services can also contribute to higher rates of relinquishment in these communities [5]. Furthermore, systemic issues, such as discrimination in housing, can disproportionately affect minority groups, leading to increased rates of pet surrender [4,7]. One study highlights that people living in lower socioeconomic areas often become pet owners passively, which can lead to higher rates of surrender when circumstances change. For instance, these individuals might acquire pets through informal means, such as finding strays or receiving them as gifts, which can result in a lack of preparedness for the responsibilities and demands of pet ownership [8]. This situation is compounded by the fact that lower-income households may face challenges such as eviction or financial hardship, making it difficult to keep pets [6].

Pets in vulnerable communities are often surrendered due to owners feeling overwhelmed by the number of animals they possess or facing challenges related to the animals’ health and behavior [9]. Behavioral issues in pets are also frequently cited as a reason for surrender. Nearly 50% of owners report behavioral problems as a contributing factor for their decision to relinquish their pets, with about a quarter identifying these issues as the primary reason [10]. Common behavioral problems include aggression, anxiety, and inappropriate elimination, which can lead to frustration for owners who may not have the resources or knowledge to address these challenges [10,11]. However, it is important to note that the reasons for surrendering pets are often multifaceted. Many owners report multiple reasons for their decision, indicating that the relinquishment process is rarely due to a single factor [2,3]. For example, while an owner may cite moving as the reason for surrender, underlying issues such as behavioral problems or financial stress may also play a significant role.

Socioeconomic factors are multifaceted and also have a connection to who seeks to adopt an animal from a shelter instead of another source. For example, educational attainment is a factor increasing the chances of an individual sourcing an animal from a shelter, particularly those with at least a college degree. This demographic tends to prioritize the reputation of the adoption source and the characteristics of the dog, suggesting a greater awareness of the importance of supporting humane organizations [12]. Families with children are often more inclined to adopt pets, although they may face barriers related to shelter requirements regarding the age of children, which can deter them from the adoption process [9]. The motivations behind pet adoption can also vary significantly among different age groups. Younger adopters, particularly those under 25, are statistically more likely to rehome their pets, indicating that age may play a critical role in the stability of pet ownership [13]. Conversely, older adopters may be more focused on altruistic motivations, such as the welfare of the animals and community benefits, rather than personal gain [14].

Moreover, the characteristics of the animals themselves, such as breed, age, and behavior, significantly influence adoption decisions. For instance, potential adopters often express preferences for specific breeds or age groups, which can vary by demographic factors such as gender and previous pet ownership experiences [15,16]. This may cause mismatches between the types of animals available at shelters and the preferences of adopters in certain demographic groups.

Beyond adoption and surrender, shelters are increasingly interested in ensuring equitable access to their services including adoption. Research by Ly (2024) and Guenther (2024) sheds light on biases within animal sheltering practices [9,17]. Ly discusses opportunities for barriers and bias during off-site animal adoption events, emphasizing the need for higher equity in services provided by animal shelters to communities [9]. Guenther delves into the legacies of the pound model in animal sheltering in the United States, pointing out issues such as institutional culture, lack of transparency, and disparities in sheltering resources across communities [17]. These studies collectively suggest that biases may exist within the operational frameworks of animal shelters, impacting the services provided and potentially influencing outcomes for shelter animals based on racial or ethnic factors. Regulations in communities that prioritize enforcement over support-based programming may lead to increased confiscation and relinquishment rates in low-income and minority communities [18,19]. Consequently, the negative experiences associated with these interactions can discourage further engagement with local government services.

Furthermore, the work of Russell-Brown (2018) on implicit racial bias highlights the prevalence of this form of bias in various contexts, including animal sheltering [20]. Implicit bias has been identified as a crucial factor influencing decision-making processes and behaviors, potentially leading to disparities in how animals are treated and adopted based on racial or ethnic characteristics of the person. This underscores the need for increased awareness and efforts to address implicit biases within animal sheltering and control operations to ensure fair and equitable treatment for all animals, regardless of their racial or ethnic associations.

This research builds upon a 2021 paper from the University of British Columbia (UBC) In this 2021 study, researchers used the Canadian Index of Multiple Deprivation (CIMD), a Canadian index with four dimensions of social vulnerability, to compare the “flow” of animals into the shelter and out for adoption across levels of vulnerability [18]. They examined animals adopted out between 2016 and 2019 from 36 SPCA shelter locations in British Columbia, Canada. The flow between the vulnerability of animals’ intake and adoption locations, grouped by quintiles, were evaluated separately for each of the four dimensions of the CIMD and for dogs, cats, kittens, and puppies. They found that three of the four CIMD dimensions were significantly different between surrendering and adopting communities (Ethnocultural Composition, Situational Vulnerability, Economic Dependency, but not Residential Instability). Due to a large sample size (*n* = 21,270) making statistical significance in the Wilcoxon Signed Rank test highly likely, they relied on effect sizes, derived as proposed by Cohen, to interpret the results [21]. These were typically small (r = 0.1–0.29) or non-existent and only once moderate (r = 0.31) for kittens and dogs under the Situational Vulnerability dimension [18].

The present study replicates the analysis in seven U.S. shelters from diverse regions and types, using the Centers for Disease and Control (CDC) Social Vulnerability Index (SVI) [22]. Since this is a different index and geography, this analysis does not intend to directly compare its results by SVI theme to the results under the four CIMD dimensions, despite some similarities. While the UBC paper was limited to animals that were owner-surrendered and then adopted, a key addition in this paper is the inclusion of animals that entered shelters as strays or were confiscated by animal control and were later adopted in the analysis. This is a critical addition because stray animals are the largest intake group entering US animal shelters, estimated at 48% of intakes nationally in 2023 and 64% of intakes of the studied shelters [23]. Also added is an analysis that compares intakes and adoptions per vulnerability quintile to the underlying distribution of vulnerability in the analyzed areas. These modifications promote a deeper understanding of the relationship between community social vulnerability, shelter intakes, and adoptions in the study communities.

This research has multiple purposes. First, we examine the social vulnerability of individuals who have either surrendered or adopted animals. We also examine the SVI of the origin of animals by intake type. These data can highlight trends in intakes and surrenders as a function of social vulnerability using a large and geographically diverse sample population. The flow between areas of low and high social vulnerability is evaluated, similar to the previous study. We also evaluate these by the various subthemes of the SVI. This challenges the idea of whether there are measurable biases in the adoption process. Lastly, the data are analyzed to identify the largest sources of animals by intake type. The overarching purpose of this work is to assist animal shelters in designing and targeting programs aimed at better serving the animals and people in their communities by illustrating the patterns that may be found in many communities in the United States.

Authors Note: We use the term “minority” throughout this paper in order to use language consistent with the CDC’s SVI subthemes. We recognize that the term ‘BIPOC’ may be preferred because there are areas where these racial/ethnic groups make up the majority of the population.

## 2. Materials and Methods

### 2.1. SVI Data

The Social Vulnerability Index (SVI) was used at the Census Tract level. The SVI is a ranked, composite index of 16 census derived variables used to assess a community’s vulnerability during times of disaster or other public health emergencies compiled and curated by the CDC [22]. The 2022 vintage SVI, the most currently available when analyzing the data, was used. The 2022 SVI is derived from data obtained from the 2018–2022 American Community Housing Survey [22]. The SVI data are provided both as an aggregate measure as well as in four different thematic indices. Due to the aggregated nature of the SVI, it is not possible to know the exact SVI of any individual household and so any analysis is subject to this limitation. The thematic composition of the SVI, with associated variables, are shown in Table 1.

### 2.2. Dataset Preparation

Data from seven shelters were analyzed. All shelters contributed their data to the Human Animal Support Services (HASS) project [24]. HASS is a national project that is aimed at improving innovation and community collaborations to improve outcomes for animals and people. There are 22 Pilot Organizations (government and non-profit animal shelters) who are active participants in HASS programs. Out of 24 shelters submitting data, these seven shelters were selected based on the quality of their geographic data, especially for stray animals. Seven regionally diverse states are represented (Missouri, South Carolina, California, Maryland, Ohio, Texas, and New York); two shelters are municipal while the others are non-profits with government contracts. Their annual intake ranges between 2000 and 10,000 animals. Some serve dense urban areas while others serve a county with several smaller cities and a more rural population. Further details about the make-up of intakes and the distribution of social vulnerability in their communities appears below. Two aggregate datasets were constructed. The first included all animals that had an adoption outcome, used to analyze the flow of adopted animals between levels of social vulnerability. The second included those animals and also all intakes that had non-adoption outcomes, used for assessing intake-related patterns only.

Cats and dogs with both an intake and an outcome in 2022–2023 were used in both datasets. The main processing step was geocoding the intake and adoption addresses to find their SVI ranking. For intake locations, the found location was used for strays and seized/custody/quarantined animals (‘Seized/Custody’ hereafter) while the owner’s address was used for owner surrenders and returns, which are analyzed as a single group for simplicity and due to the blurry lines between the two across shelters. Transfer-ins, animals entered temporarily (typically classified under Clinic or Service In) and animals born in care were not included in the analysis for having no relevant intake location, leaving 51,994 animals with relevant intake types. For adoptions, the adopter’s address was used. Adopted animals had both their intake and adoption address geocoded, while animals with other outcomes (e.g., return to home, transfer out, euthanasia) had only their intakes geocoded. All addresses were first geocoded and then mapped onto a Census Tract to find their SVI ranking. Geocoding was carried out through a combination of the Google Maps API and PositionStack API. Addresses were first geocoded through the latter, and if the result was unsuccessful (failure or lower than 100% confident result) the former was used. Animals with intake or adoption addresses that failed with both geocoding services (either for having an incomplete location to start with or despite having an address that looked complete) were removed from analysis (*n* = 8014). Additionally, the distance of successful results from the shelter locations was examined and 992 animals with addresses that were more than 300 miles away from the shelter were treated as outliers and removed from analysis.

The intake and adoption addresses were then assigned a Census Tract using the tidycensus package in R (version 4.2.2). Census Tracts based on the 2022 American Community Survey were used. The Census Tract was then used to match an SVI ranking, which was converted to quintiles, for ease of comparison with previous research. Animals with addresses that failed to be assigned a Census Tract or an SVI ranking (some Census Tracts were missing in the SVI files, for example) were removed (*n* = 697).

The complete adoption dataset included 42,291 animals from the seven shelters combined, which were all processed similarly—had both intake and adoption addresses successfully geocoded, kept after outlier adoptions (over 300 miles), and mapped to a Census Tract and SVI ranking. This amounts to 81% of all relevant adoptions (51,994), i.e., animals that entered the shelter through relevant intake tracks (not transfer-ins, animals born in care, etc.) and were later adopted. A further 43,224 animals with non-adoption outcomes whose intake address was successfully geocoded were used when analyzing only intake-related patterns, combined with the adopted animals for a total of 85,515 animals.

### 2.3. Analysis

All data analysis was performed in R (version 4.2.2). To provide context for the analysis, the distribution of SVI quintiles of all Census Tracts in the dataset was summarized using a stacked bar chart for the overall SVI ranking and each of the SVI themes. The correlation between the SVI ranking themes was also found using Pearson’s correlation and summarized in a matrix.

The change between intake and adoption SVI quintile was visualized through alluvial diagrams, first for the entire first dataset, and then separately for the main intake types (Stray, Owner Surrenders and Returns, and Seized/Custody) and age/species (combinations of Dog/Cat and Adult/Youth). Animals whose age at intake was 180 days or younger were classified as Youth (sometimes referred to as puppies or kittens later), and all others were classified as Adults. To examine the contribution of the various SVI themes to the observed trends, the alluvial diagram for the entire dataset was produced four times, each using a different SVI theme instead of the overall SVI ranking.

To complement the visual comparison, intake and adoption SVI scores were compared using Wilcoxon Signed Rank Tests. Tests were performed for the whole dataset with the different SVI themes, for each intake type separately, and for age/species combinations. Because a large sample size can produce very small *p*-values, effect sizes were also calculated for each test by dividing the test statistic by the square root of the sample size [25]. Following the proposed standard for interpreting effect sizes, 0.10–0.29 was considered a small effect, 0.30–0.49 moderate and ≥0.5 large [21]. The tests results and effect sizes were all produced using the implementation of the tests in the rcompanion R package.

The second part of the analysis was performed using the larger dataset that included intakes that were not adopted for questions regarding intake. The two ‘sides’ of the alluvial diagrams (the distribution of intake and outcome SVI) were compared separately to the underlying distribution of SVI ranking by dividing intakes and adoptions by the total number of households across all Census Tracts in each SVI quintile that had at least ten intakes and adoptions combined, multiplied by 1000 for clarity of presentation. The normalized intakes-per-1000-households and adoptions-per-1000-household numbers for each SVI quintile were visualized through a bar chart to show the differences in SVI levels between animals coming into the shelter (regardless of outcome) and adoptions (regardless of intake SVI) while accounting for the variation in the number of households grouped under each SVI quintile. These figures are then reproduced for each intake type separately to highlight the relative volume of intakes from different sources, which becomes obscured by the alluvial diagrams.

For the five of the seven shelters that collected informative intake reasons (i.e., not empty or general) for owner surrenders and returns, the differences in distribution of reasons between intake SVI were also examined. Only one intake reason was entered for each animal at all shelters. For this part, intake reasons were coded into six standard categories based on the values entered by the shelter: Behavior (any value including behavior, training or aggression), Cost (values indicating not being able to afford some component of care), Medical (values including health of animal, injured, sickness), Housing (landlord issues, homelessness, moving and similar values), Permanent Change (divorce, new child, owner death, deployed abroad and alike) and Preferences/Time (values indicating compatibility issues with children or existing pets, allergies, inadequate housing situations, and alike). General values (like “other” and “general”) were removed (1666 animals), as well as all other values, which were no more than 10% of each shelter’s given reasons. After filtering, 9391 animals were included, having an informative intake reason (i.e., not empty, “general”, etc.), out of 12,003 Owner Surrender/Returns intakes in the dataset. The distribution of reasons within each SVI quintile was visualized through a stacked bar chart.

Finally, the distribution of outcome types within each intake SVI quintile were compared, to assess whether animals coming from different levels of vulnerability have different outcomes. Outcome types included Adoption, Transfer Out, Return to Owner, and Euthanasia. Other outcome types—Trap, Neuter, Release or Shelter, Neuter, Release (TNR/SNR), Lost/Stolen, and Died in Care—were excluded because they accounted for less than 3% of outcomes. The distribution of outcome types within each SVI quintile was visualized through a stacked bar chart.

## 3. Results

### 3.1. Contextual Results

Table 2 shows the total number of records for each shelter in both aggregate datasets, broken down by intake type. When looking only at adopted animals (left-hand side), two of the shelters had a fairly small share of the dataset (7% combined), and unlike the two other intake types, Seized/Custody intakes were primarily from three of the seven shelters (1, 2, and 5). When looking at all intakes (right-hand side), both these patterns are not as pronounced.

**Figure 1:**
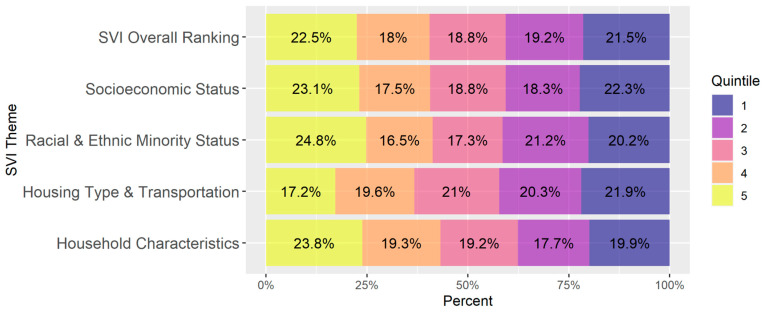
Breakdown of quintile distribution at the Census Tract level of all counties within the combined study communities. The figure indicates the relative even distribution of the population across SVI quintiles. Note: Quintile 1 is least vulnerable and 5 is the most vulnerable.

The breakdown of SVI quintiles in all study communities in aggregate is roughly uniform, as shown in Figure 1.

**Figure 2:**
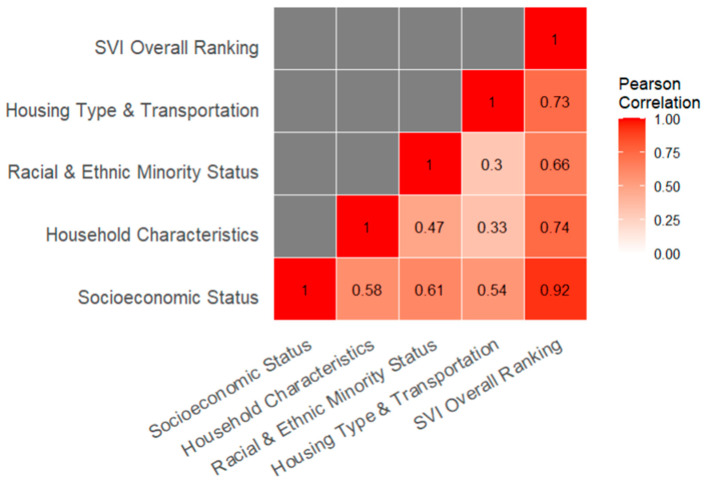
Correlation between SVI themes across all Census Tracts.

Figure 2 shows Pearson’s R between all pairs of SVI themes for the first aggregated dataset (only using intakes which were later adopted).

Table 3 shows the number of animals under each intake type and species group in the 2nd dataset (including all intakes).

### 3.2. Intake to Adoption Flow, Overall and by Intake Type

**Figure 3:**
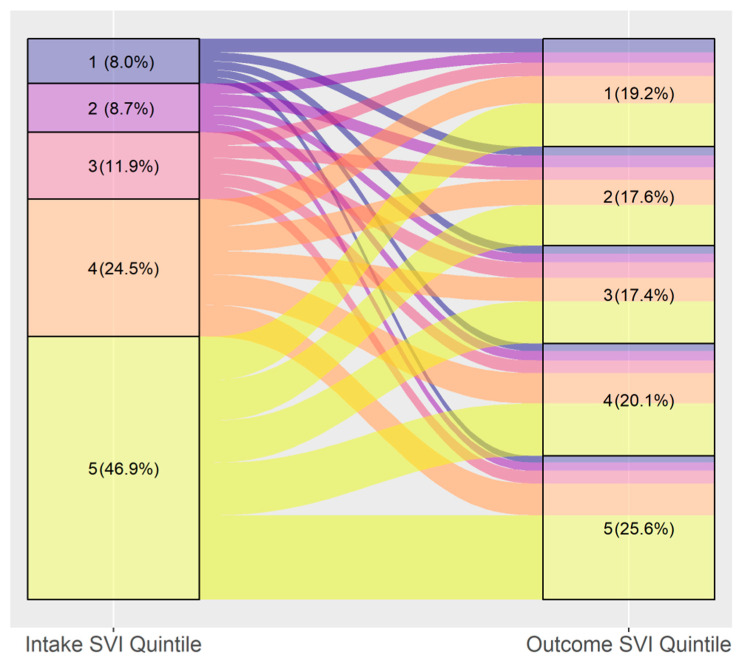
Flow of animals from intake to adoption by SVI quintile (*n* = 42,291). Quintile 1 is the least vulnerable and 5 is the most vulnerable.

Figure 3 shows the overall flow of animals from SVI quintile at intake to SVI quintile of the adopter. Animals tended to enter the shelter from areas of higher vulnerability, and are adopted at relatively similar rates across the different SVI quintiles. In total, 47% of intakes are from the most vulnerable quintile, which accounts for 22.5% of the surveyed Census Tracts, while only 8% came from the lowest-vulnerability areas, which in a proportionate distribution would be 21.5%.

**Figure 4:**
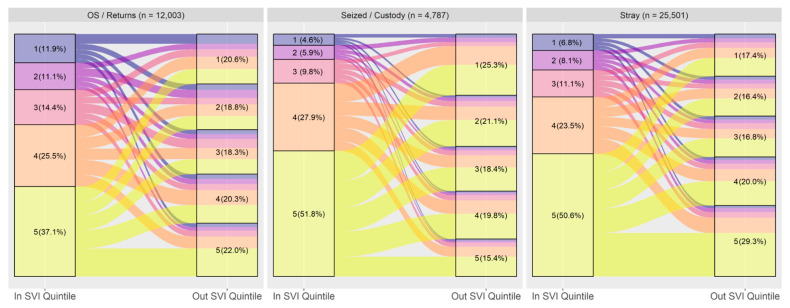
Flow of animals from intake to adoption by SVI quintile, divided by intake type (*n* = 42,291). Quintile 1 is least vulnerable and 5 is the most vulnerable. Note ‘OS’ denotes Owner Surrender.

Figure 4 shows the alluvial diagram divided into the three main intake categories—owner surrenders/returns, seized or confiscated animals, and stray intakes. The trend of animals flowing into the shelter from higher vulnerability areas and out of the shelter into lower vulnerability areas was more pronounced for animals coming in as seized or confiscated or as strays than animals surrendered by their owners.

Table 4 summarizes Wilcoxon Signed Rank Test results comparing intake and adoption overall SVI score in the entire dataset and divided by intake type. It validates what can be visually seen in Figure 4—the disparity between SVI at intake and adoption is strongest for animals that have been seized or confiscated, though there is still a statistically significant moderate effect on strays and owner surrendered animals. The effect is weakest for owner surrendered animals.

### 3.3. Overall Flow Using Different SVI Themes

**Figure 5:**
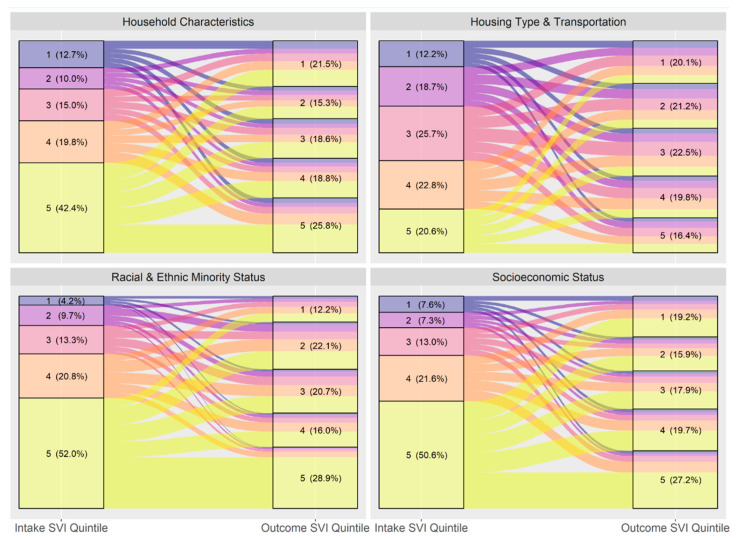
Flow of animals from intake to adoption by SVI quintile, rotating SVI themes (*n* = 42,291). Quintile 1 is least vulnerable and 5 is the most vulnerable.

Figure 5 shows the overall intake SVI to adoption SVI quintiles like Figure 3, but rotating between the four SVI themes instead of using the overall ranking. Table 5 then shows the Wilcoxon Signed Rank Test results for each SVI theme used in turn. The largest effect size was found when the measure of vulnerability was the Racial and Ethnic Minority Status.

### 3.4. Overall Flow by Age and Species

**Figure 6:**
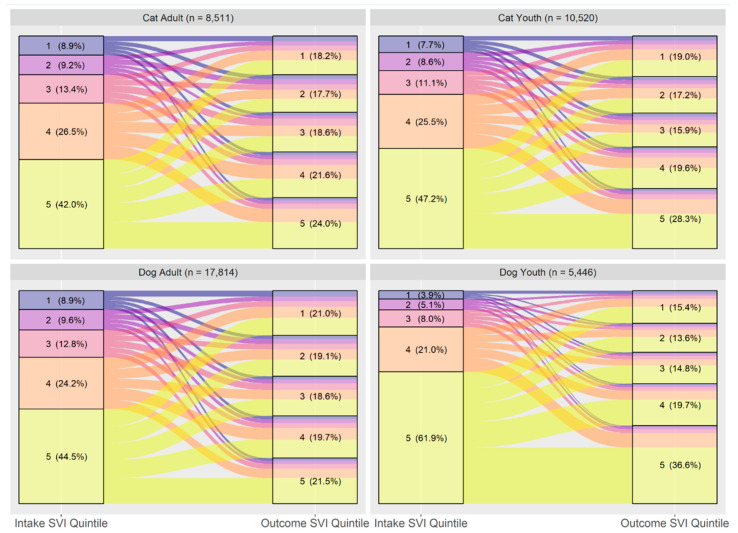
Flow of animals from intake to adoption by SVI quintile, divided by age and species (*n* = 42,291). Quintile 1 is least vulnerable and 5 is the most vulnerable.

Figure 6 and Table 6 shows the flow diagram and Wilcoxon Signed Test results broken down by a combination of species and age group. Differences between age groups within species and vice versa are minor.

### 3.5. Intakes and Adoptions per 1000 Households (2nd Dataset)

**Figure 7:**
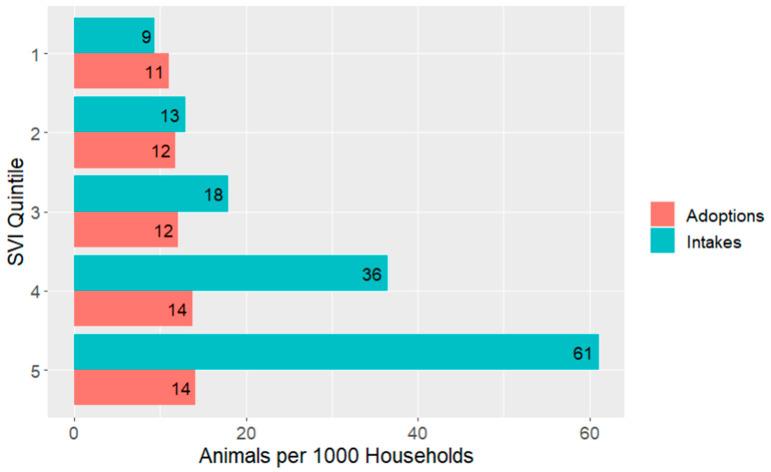
Intakes and adoptions per 1000 households within SVI quintiles. Quintile 1 is least vulnerable and 5 is the most vulnerable.

As another way of exploring the data, Figure 7 shows the intakes and adoptions per 1000 households for each of the SVI quintiles. Intakes-per-household, which include all intakes and not only those adopted (the 2nd dataset described above), increase with each level of vulnerability, while adoptions per household (which include only adopted animals, as shown in the alluvial flow diagrams) did not vary with vulnerability levels.

**Figure 8:**
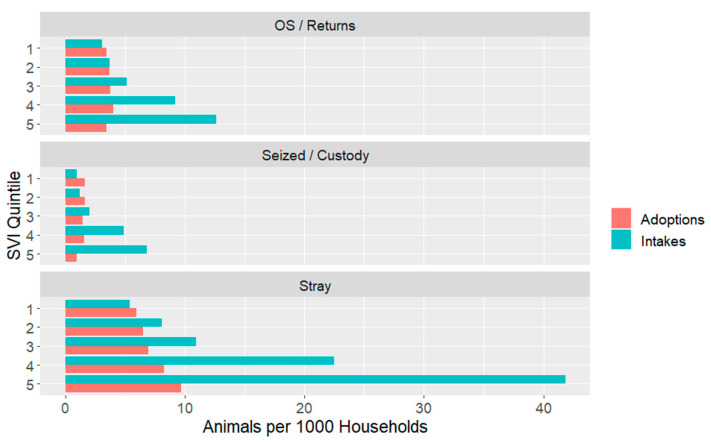
Intakes and adoptions per 1000 households within SVI quintiles, divided by intake type. Quintile 1 is least vulnerable and 5 is the most vulnerable. OS refers to Owner Surrender.

This trend is also reflected in Figure 8, in which all intakes and adoptions are separated by intake type. This figure also reflects the volume difference between the categories. All intake types demonstrate disproportionate intake from the highest-vulnerability areas, but in relative terms, the disparity is strongest for Seized/Custody and stray intakes, while the latter also account for most animals. Adoptions are again not different by the adopter’s vulnerability level.

### 3.6. Intake Reasons and Outcomes by Intake SVI Quintile

**Figure 9:**
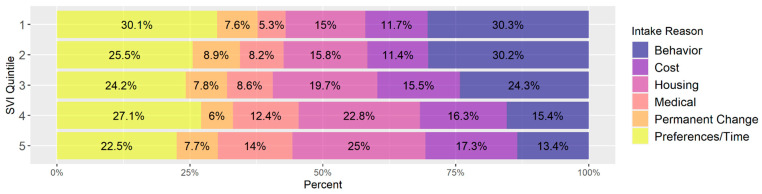
Distribution of intake reason groups within each intake SVI quintile. Quintile 1 is least vulnerable and 5 is the most vulnerable.

Figure 9 shows the breakdown of intake reasons for all owner-surrendered or returned animals (not only those adopted) within each intake SVI quintile, for five of the seven shelters that collected informative intake reasons (all but Shelters 3 and 6). Key patterns include higher percentages of behavior and time/preference owner surrenders and lower percentages of cost, medical, and housing-related owner surrenders among lower vulnerability communities compared to higher vulnerability ones.

**Figure 10:**
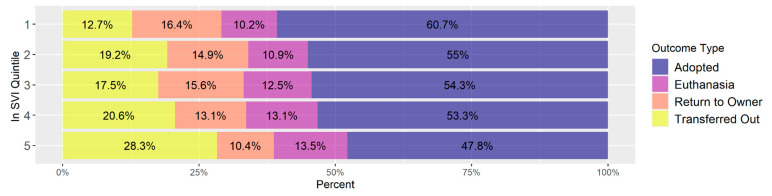
Distribution of outcomes within intake SVI quintiles. Quintile 1 is least vulnerable and 5 is the most vulnerable.

Figure 10 shows the breakdown of outcome types among all intakes by Intake SVI. Euthanasia and Return to Owner rates are fairly consistent across intake SVI quintiles. In higher SVI quintiles more animals were transferred out and fewer animals were adopted.

## 4. Discussion

In general, our findings show that a predominance of animals entering the shelter system originate from areas that have the highest levels of social vulnerability as measured by the SVI. Our research yielded different results than the Canadian study though direct comparison is difficult given that the measure of vulnerability is different between the two countries. We found that the largest effect sizes in differences between intake and adoption vulnerability were in the Racial and Ethnic Minority subtheme (see Table 5) (r = 0.49) closely followed by the Socioeconomic Status (r = 0.42), both of which had Moderate effect sizes. Household Characteristics (r = 0.29) and Housing Type and Transportation (r = 0.17) were statistically significant but the effect size was small. It is worth noting that there is moderate correlation (Pearson’s R of 0.61) between the Racial and Ethnic Minority subtheme and the Socioeconomic subtheme suggesting that there is an intersectionality of social vulnerability between these two subthemes. Due to the large dataset, the effect sizes of the tests assessing the differences between intake and adoption SVI are most helpful for interpreting the results. The overall SVI ranking was less strong (r = 0.41) than Racial and Ethnic Minority status though it remains an important tool for general community assessments in sheltering. The skew towards higher vulnerability at intake than adoption for all intake types under the overall SVI and both Socioeconomic Status and Racial and Ethnic Minority Status in particular was stronger than the one found in British Columbia, as measured by the effect sizes, especially for stray (r = 0.4) and Seized/Custody (r = 0.63) animals.

### 4.1. Intakes

Consistently, intake SVI levels were skewed so that more intakes came from areas of higher vulnerability, even when accounting for the number of households in those areas. This pattern was observed across the individual shelters we studied with variances only in the magnitude of the difference and is magnified after normalizing intakes by the number of households at each SVI level in the communities. For example, the highest quintile of SVI represented 47% of the intakes despite this quintile only representing 22% of the overall study population. This is largely driven by specific subsets of intake categories, specifically stray and Seized/Custody animals. Stray dogs and cats comprised over 60% of the total intakes in the study communities with seized or confiscated animals comprising nearly 13% of the total intakes, making this finding particularly important for targeting programs designed to reduce shelter intakes overall. These findings clearly indicate that programs focused on reducing the intake of stray animals from regions of highest social vulnerability would have the greatest potential impact on overall shelter intakes.

Owner-surrendered animals had a smaller effect size, indicating a weaker (but still present) disparity between intake and adoption SVI. Shelter intake prevention programs often highlight the importance of providing support for low-income individuals who may struggle with keeping their animals due to challenges affording pet food, added rental fees associated with having a pet or the rising cost of veterinary care. However, in our study communities, owned animal intakes represented only about a quarter of the total intake volume. Areas of higher social vulnerability were more likely to have owner-surrendered animals, but the effect was not as strong as it was for stray and confiscated animals. It is possible that this reflects the success of community safety net programming already existing within the sampled shelters that made up this study.

This does not imply that a large number of owned animals are not being rehomed in areas of high social vulnerability. For example, previous findings indicate that dogs in lower-income homes were substantially more likely to be given away to friends or family members as opposed to being surrendered to an animal shelter [26]. While this effect was also observed at higher levels of income, there was a steady trend toward increasing probability that dogs leaving the home would be taken to the shelter versus given away as household income increased [26]. It is, however, an important finding for shelters looking to evaluate the impact of community safety net programming. The amount of rehoming happening outside of the shelter system means that programs aimed at supporting low-income homes in keeping their animals may not result in a large impact on the number of animals actually surrendered to the shelter. Thus, impact evaluation plans should not rely entirely on owner surrender intake reduction as the sole metric. Additionally, since the populations of truly unowned, free-roaming animals are probably fairly small, many of the animals entering the shelters as “strays” could have been previously cared for, suggesting that programs targeted at supporting struggling pet owners could affect stray intakes as well.

### 4.2. Surrender

There were also noticeable differences in the reasons owned animals were surrendered to the shelter as a function of SVI. These differences can be difficult to interpret, however, as only a single reason is provided for intakes and so may not fully reflect the complex reasons that individuals choose to surrender an animal. Animals originating from homes of high social vulnerability were more often surrendered for reasons related to housing, as seen in Figure 9. This is consistent with previous research that has found more restrictive policies around companion animals in rental housing in areas with larger minority populations than in properties in predominantly White areas [7]. There was also a trend toward more animals in higher SVI areas being surrendered for reasons associated with cost and medical needs. These findings suggest that programs focused on increasing the availability of pet-friendly housing options in areas of high social vulnerability may be the most targeted way of preventing owner surrender of animals to shelters with material support being the next most impactful. As SVI decreases, surrenders for reasons of behavior or the individual preferences/time availability of owners become more important. This suggests that a more complex intervention is needed in communities with less social vulnerability that could focus on behavior modification resources.

Additionally, these results do not imply that people living in higher-vulnerability areas are less responsible pet-owners or less connected to their pets, with previous research also suggesting otherwise [27,28,29]. Rather, it is possible that people living in higher-vulnerability areas are simply facing challenges keeping their pets related to their cost of care, housing, or medical situation, as reflected by the differences in intake reasons bringing people to surrender. Another significant finding is that the Racial and Ethnic Minority status had the largest effect size, especially high for Seized/Custody animals. A high ranking in this sub theme simply reflects a large non-White population, and previous research in human policing implying the potential that such areas are over-enforced and over-policed [30,31]. Hawes and colleagues (2020) suggested in commentary that animal control agencies should examine if the enforcement of their policies is laden with implicit bias rather than recognizing the role of structural inequities in driving both human and animal welfare [19]. Previous research by Companions and Animals for Reform and Equity (CARE) has indeed identified negative attitudes within the animal welfare industry toward Black and low-income animal owners [32]. These results may reflect disproportionate enforcement in low-income and non-White communities. It is equally possible that these results indicate the reality of animal welfare in communities with complex socioeconomic challenges. Previous research in Australia, for example, found that dog welfare complaints were more common in impoverished areas, particularly with complaints related to cruelty and neglect [33]. In both cases, the results demonstrate clearly how socioeconomic circumstances shape shelter intake, suggesting that interventions intended to target the latter would benefit from examining the former.

Another insight generated relates to the age of the animals surrendered/found and the SVI. Unlike the UBC analysis, the effect sizes for the flow of puppies and kittens was similar to that of adult cats and dogs. As shown in Figure 6, puppies originating from areas in the highest quintile of the SVI represent about 62% of puppies incoming to the study shelters compared to only about 45% of adult dogs. This implies that activities aimed at promoting and providing access to spay and neuter would best be targeted in Census Tracts of communities in the lowest quintile of the SVI. A similar trend is seen with respect to the SVI of origin with nearly 73% of kittens coming from the lowest two quintiles of social vulnerability. As reported in Table 3, dogs represented approximately 64% (*n* = 54,898) of the total intakes in the study shelters compared to 36% (*n* = 30,617) being cats. However, of cat intakes, 52% are kittens while of dog intakes 24% are puppies in the study shelters. With the large portion of kittens incoming to shelters, particularly from areas in the highest two quintiles of SVI, these results would indicate that programs aimed at providing sterilization for cats, particularly free roaming cats, would have the greatest potential impact on intake reduction as also indicated by previous research [34,35]. The need for spay neuter programs in low-income communities is further evidenced by the large numbers of young animals that are stray intakes. As seen in Table 3, the majority of kittens and puppies (73%) are entering the study shelter systems as strays compared to entering as owner surrender or confiscation.

Implementation of sterilization programs can be complicated as multiple factors have been found to influence animal sterilization rates. Studies have indicated that low-income families may have a lower percentage of cats being neutered compared to higher-income households [36]. Additionally, research has shown that Hispanic residents in certain communities may have lower rates of sterilization for their pets, with only a small percentage of dogs and cats being sterilized despite positive attitudes toward the practice indicating other barriers to receiving the care exist [37,38]. Furthermore, a study in Brazil found that only a minority of animals were sterilized, with socioeconomic factors playing a role in the decision to spay or neuter pets [39]. Ensuring affordable and culturally competent access to veterinary services is also central to addressing this issue [40,41].

These results also provide additional evidence that community cat programs aimed at sterilizing and returning cats to the locations where they were found through trap-neuter-vaccinate-return (TNVR) may have an important social equity underpinning. Similarly, shelters that do not provide this service but want to provide more services to marginalized communities should consider the targeted implementation of such programs in areas with high levels of social vulnerability. Individuals who feed and care for community cats have been shown to represent the general diversity of their community, and to be as attached to the cats they care for as individuals who own cats [42,43].

### 4.3. Outcomes

In general, the patterns for animal outcomes are similar regardless of their originating SVI as shown in Figure 10. In other words, animals that came from areas of higher social vulnerability were not euthanized at higher rates than animals originating from less vulnerable populations in the community. If we assume that higher euthanasia implies more medical and behavioral problems, it is interesting to see this pattern despite knowing that individuals in high social vulnerability communities may have more challenges accessing medical and behavioral professionals. It is important to note, however, that among these shelters, the overall euthanasia rates are relatively low and the time and expense to treat medical or behavioral interventions needed to progress animals to the point they can be adopted is not measured in these data. One additional point to clarify in Figure 10 is the disproportionate number of animals transferred out that originate from the areas of highest social vulnerability. This is largely due to a single shelter that had high levels of transfers that also was in a majority high vulnerability community and does not reflect a greater trend among the entire group of study shelters.

The distribution of where animals are adopted paints a more equitable distribution and may upend some assumptions about who adopts animals from shelters (at least shelters included in this study). This is easiest to observe in Figure 7 where the adoptions have been normalized per 1000 households in each of the SVI quintiles. There is no trend apparent at all to indicate the individuals of higher social vulnerability are any less likely to adopt an animal from a shelter versus residents in areas of lower social vulnerability. This implies that, at least in the shelters that comprised this study, barriers to adoption are not experienced at higher rates by individuals of high SVI. Past research indicated that lower-income individuals were more likely to obtain their dogs from friends or family versus a shelter, which may indicate one of two things [26]. First, it possibly implies that there is something unique about these two different sample populations or simply it may reflect a high volume of animal transience in areas of higher SVI with a higher volume of animals being rehomed than in areas of lower SVI. Additionally, the shelters included in this study may be performing more active community safety net programming and employing more progressive adoption processes than typical shelters, which could have reduced previous barriers to adoption and contributed to the uniform pattern in adoption vulnerability levels.

### 4.4. Limitations and Future Research

There are a few notable limitations to this research. Our study only had detail for individual animal shelters operating in their community and so does not necessarily paint a holistic picture of patterns in SVI and overall animal sheltering in each area examined. For example, some communities have multiple animal shelters serving the same general area, and only three of the shelters had a large volume of Seized/Custody animals. Future research that uses datasets from all animal shelters in a community would provide a more complete evaluation. Further research exploring enforcement patterns could also shed light on whether the patterns are simply reflective of the reality in certain communities, indicate areas in need of additional material support, or reflect an institutional bias. The shelters in our study were also all part of the HASS program. It is possible that different patterns would be seen in other shelters that operate with a different set of shelters not associated with this program. Our study also only represents a small number of communities. Given the large variance in socioeconomics, culture, and operational priorities our results may not represent national trends in sheltering. The data for SVI is only available at the smallest geometry of the Census Tract, and so it does not necessarily accurately represent any given household within the tract. Lastly, are the challenges inherent in animal shelter data. Intake reasons are also notoriously difficult to interpret and are inherently a single data point for what is typically a multifaceted decision. Data on the location of stray animals can be difficult to geocode due to nonspecific locations. Given the large numbers of stray animals that shelters take in, future research that provides a deeper understanding of whether these animals are truly stray could also add insight to our knowledge of how to improve outcomes for people and animals in the community.

## 5. Conclusions

Challenges in animal sheltering continue as shelters work to support their communities, reduce intakes, and increase adoptions. This research has shed light on the patterns of animal intakes and adoptions as a function of social vulnerability, as measured by the SVI. Disproportionate numbers of animals originate from areas in the lowest quintile of the SVI. This is particularly profound for animals entering the shelter due to being found as strays or from seizure/confiscation. This highlights the need for developing a further understanding of the circumstances resulting in stray animals, particularly stray adult dogs in communities with the highest social vulnerability. The work also underlines the need for more spay and neuter programs in areas in the highest SVI including Trap-Neuter-Vaccinate-Return programming for community cats. Adoptions are much more evenly distributed among the SVI groups, which indicates that shelters in the study communities are consistently engaging with individuals from various socioeconomic backgrounds in the adoption process.

## Figures and Tables

**Table 1:** Variables comprising each subtheme of the SVI based on the 2022 data definitions provided by the CDC.

Theme	Variables
	Below 150% Poverty
Socioeconomic Status	Unemployed
	Housing Cost Burden
	No High School Diploma
	Aged 65 and Older
	Aged 17 and Younger
	Civilian With a Disability
Household Characteristics	Single-Parent Households
	English Language Proficiency
Racial and Ethnic Minority Status	Non-White Hispanic and All Other Non-White Races/Ethnicities
	Multi-Unit Structures
	Mobile Homes
Housing Type and Transportation	Crowding
	No Vehicle
	Group Living Quarters

**Table 2:** Number of animals by intake type included in the analysis from each shelter.

	Animals Adopted				All Intakes			
Shelter	Stray	Owner Surrender/Returns	Seized/ Custody	Total	Stray	Owner Surrender/ Returns	Seized/Custody	Total
Shelter 1	3769	3005	1366	8140 (19.2%)	6535	5234	2797	14,566 (17%)
Shelter 2	3709	982	676	5367 (12.7%)	6623	1248	1256	9127 (10.7%)
Shelter 3	1092	233	10	1335 (3.2%)	8211	1683	155	10,049 (11.8%)
Shelter 4	4198	1295	15	5508 (13%)	8189	2259	100	10,548 (12.3%)
Shelter 5	5781	4794	2618	13,193 (31.2%)	8584	6136	4867	19,587 (22.9%)
Shelter 6	5667	1317	85	7069 (16.7%)	13,684	3152	270	17,106 (20%)
Shelter 7	1285	377	17	1679 (4%)	3191	1220	121	4532 (5.3%)
Total	25,501	12,003	4787	42,291 (Total)	55,017	20,932	9566	85,515 (Total)

**Table 3:** Number of animals entering shelters under the three main intake types analyzed by species and age group. Note the abbreviation ‘OS’ denotes Owner Surrender.

Species + Age Group	OS/Returns	Seized/Custody	Stray	Total
Cat Adult	5184 (35.6%)	1313 (9%)	8061 (55.4%)	14,558
Cat Youth	3770 (23.5%)	619 (3.9%)	11,670 (72.7%)	16,059
Dog Adult	9343 (22.4%)	6680 (16%)	25,741 (61.6%)	41,764
Dog Youth	2635 (20.1%)	954 (7.3%)	9545 (72.7%)	13,134
Total	20,932 (24.5%)	9566 (11.2%)	55,017 (64.3%)	85,515

**Table 4:** Wilcoxon Signed Rank Test results for intake and adoption SVI rankings by intake type.

	Number of Animals	Test Statistic (V)	*p*-Value	Effect Size	Interpretation
The full dataset	42,313	618,336,419	<2.2 × 10^−16^	0.41	Moderate
Owner Surrender and Returns	12,003	43,276,451	<2.2 × 10^−16^	0.34	Moderate
Strays	25,501	228,899,532	<2.2 × 10^−16^	0.4	Moderate
Seized/Custody	4787	9,699,958	<2.2 × 10^−16^	0.63	Large

**Table 5:** Wilcoxon Signed Rank Test results for intake and adoption SVI rankings by SVI theme.

SVI Theme	Test Statistic (V)	*p*-Value	Effect Size	Interpretation
Overall ranking	618,336,419	<2.2 × 10^−16^	0.41	Moderate
Socioeconomic Status	621,435,795	<2.2 × 10^−16^	0.42	Moderate
Household Characteristics	561,214,670	<2.2 × 10^−16^	0.29	Small
Racial and Ethnic Minority Status	651,031,509	<2.2 × 10^−16^	0.49	Moderate
Housing Type and Transportation	502,053,477	<2.2 × 10^−16^	0.17	Small

**Table 6:** Wilcoxon Signed Rank Test results for intake and adoption SVI rankings by age and species.

Group	Test Statistic (V)	*p*-Value	Effect Size	Interpretation
Dog Adult	109,505,241	<2.2 × 10^−16^	0.43	Moderate
Dog Youth	10,807,224	<2.2 × 10^−16^	0.45	Moderate
Cat Adult	23,791,960	<2.2 × 10^−16^	0.37	Moderate
Cat Youth	38,824,039	<2.2 × 10^−16^	0.39	Moderate

## Data Availability

The data used in this study are not available due to the need to protect individual and organizational privacy.
